# Nasal Rinsing with Probiotics—Microbiome Evaluation in Patients with Inflammatory Diseases of the Nasal Mucosa

**DOI:** 10.3390/jcm14103341

**Published:** 2025-05-11

**Authors:** Eliza Brożek-Mądry, Laura Ziuzia-Januszewska, Oliwier Misztal, Zofia Burska, Ewelina Sosnowska-Turek, Janusz Sierdziński

**Affiliations:** 1 Department of Otorhinolaryngology, National Institute of the Ministry of the Interior and Administration, 02-507 Warsaw, Poland; 2 Faculty of Chemistry, University of Warsaw, 02-093 Warszaw, Poland; 3 Probios, 10-683 Olsztyn, Poland; 4 Department of Medical Informatics and Telemedicine, Medical University of Warsaw, 00-581 Warsaw, Poland; janusz.sierdzinski@wum.edu.pl

**Keywords:** microbiome, microbiota, chronic rhinosinusitis, granulomatosis with polyangiitis, nasal septal perforation, probiotics, nasal rinsing

## Abstract

**Background/Objectives**: The evidence regarding the efficacy of probiotics in chronic rhinosinusitis (CRS) is very limited, prompting the EPOS2020 steering group to advise against their use in CRS treatment. Therefore, further research to evaluate the impact of probiotics on microbial communities is particularly important. This study aimed to assess the influence of probiotic nasal rinses on nasal microbiota profiles in patients with primary CRS, granulomatosis with polyangiitis (GPA), and nasal septal perforation (NSP) using 16S rRNA sequencing. **Methods**: Thirty-six patients with nasal mucosal diseases, including sixteen with primary CRS, eleven with GPA, and nine with NSP, were randomly assigned to either a study group receiving nasal rinses with probiotics containing *Lactobacillus plantarum* and *Bifidobacterium animalis*, or a control group using nasal rinses with saline. Metagenomic analysis targeting the V3–V4 hypervariable region of the 16S rRNA gene was performed to characterize bacterial and archaeal populations. **Results**: At the genus level, the most abundant co-colonizers included *Staphylococcus*, *Streptococcus*, and *Haemophilus.* After one month of probiotic rinsing, a decrease in abundance of the genera *Finegoldia* (*p* = 0.010), *Haemophilus* (*p* = 0.020), *Streptococcus* (*p* = 0.027), *Staphylococcus* (*p* = 0.033), *Micrococcus* (*p* = 0.035), *Corynebacterium* (*p* = 0.049), *Gemella* (*p* = 0.055), *Rubrobacter* (*p* = 0.055), and *Pseudonocardia* (*p* = 0.058) was observed. Conversely, the abundance of probiotic species *Lactobacillus plantarum* and *Bifidobacterium animalis* increased. Moreover, increases in the genera *Dolosigranulum* and *Stenotrophomonas* were observed, although they did not reach statistical significance. **Conclusions**: Probiotic nasal rinses may contribute to restoring microbial homeostasis by reducing genera associated with inflammatory dysbiosis in nasal inflammatory diseases, warranting further research on their clinical benefits.

## 1. Introduction

Chronic rhinosinusitis (CRS) is a heterogeneous sinonasal inflammatory disease that affects 5–12% of the general population, causing a significant global socioeconomic burden. The pathophysiology of CRS is multifactorial, with the main hypothesis based on diverse molecular pathways or endotypes driven by a dysfunctional host response against pathogens’ breach of the mucosal barriers [1,2,3].

In a healthy population, sinonasal mucosa serves as a relative barrier regulating interactions of environmental agents with the host immune system and contributing to the development of tolerance, competitive inhibition of pathogens, and generation of important metabolites. Mucosal breach results in a cellular and cytokine immune response, which physiologically aims to eliminate the pathogens and restore barrier integrity [1,4].

However, in CRS, this immune response is dynamic, heterogeneous, and polyclonal, targeting antigens derived from multiple organisms and does not self-limit. Moreover, it is often associated with various types of tissue remodeling, such as polyp formation, goblet cell hyperplasia, fibrosis, and epithelial barrier abnormalities, resulting in its greater permeability [1].

Traditionally, CRS has been classified into chronic rhinosinusitis with nasal polyps (CRSwNP) and without nasal polyps (CRSsNP). However, as understanding has been gained over time that CRS is a complex and broad syndrome with different pathophysiologies, the main emphasis in its classification has been on the predominant endotype in the underlying inflammatory response [1,4,5]. The European Position Paper on Rhinosinusitis and Nasal Polyps 2020 (EPOS2020) distinguishes primary and secondary CRS, each divided into localized and diffuse diseases based on anatomical distribution [1]. Primary CRS is further classified based on the endotype dominance into either type 2 or non-type 2. In secondary CRS, further classification depends on local pathology, as well as mechanical, inflammatory, and immunological factors [1]. Thus, the molecular pathways causing mucosal inflammation and tissue remodeling are hypothesized to be driven by dysfunctional interactions at the mucosal surface between the host and the environmental agents, such as viruses, bacteria, fungi, allergens, pollutants, and cigarette smoke, with microbiota generally considered as the most important environmental drivers of CRS [1].

Importantly, advances in molecular techniques resulted in a shift away from the paradigm of the presence of a single pathogen as the source of disease. Indeed, standard culture methods offer only a limited range of conditions for microbial growth and, therefore, omit taxa that are non-culturable or difficult to culture [1,6,7]. The introduction of culture-independent microbial DNA sequencing techniques, such as targeted sequencing of bacterial 16S ribosomal RNA (16S rRNA) marker gene and shotgun sequencing of the metagenome, has allowed for a better characterization of human microbiota [1,7,8,9]. This has enabled the development of the microbiome concept, in which microbiota inhabiting the human body surfaces create an ecological community remaining in a complex network of interactions, both within it and with the host organism, and playing a key role in maintaining normal human physiological, metabolic, and immunological functions [4,6,9,10]. The microbiome plays a crucial role in the development of the host mucosal barrier function, mucus production, prevention of colonization and proliferation of pathogens, as well as in innate and adaptive immune response [6,9,11].

Various factors, including age, genetic susceptibility, and environmental factors, such as infections, lifestyle, and medications, may lead to an imbalance in a microbial composition called dysbiosis. Since the microbiome affects numerous processes that may be implicated in the pathogenesis of CRS, the dysbiosis hypothesis has been suggested as a potential mechanism involved in the development of this disease [1,3,4,7,11,12]. For this reason, determining the relationship between the composition of the nasal and paranasal sinus mucosa microbiome and the presence and severity of CRS may increase our understanding of the pathobiology of this complex syndrome [1,7,12,13].

Notably, there is considerable variation in the results of studies on the sinonasal microbiome. These discrepancies may be due to a limited number of subjects; diverse sampling methodology and technical approaches; different anatomical sampling sites, which affects the results due to intraindividual differences in distinct sinonasal microenvironments; population differences with various confounding factors such as age, comorbidities, and genetic background of patients; severity and phenotype of CRS, as well as prior treatments, including surgery or recent antibiotic use; and most importantly, high inter-individual variability [1,3,6,7,9,14,15].

A large variance between studies highlights the need for larger, controlled studies with a unified methodology [1,3,8]. In addition, the most frequently used culture-independent techniques, such as 16S rRNA gene sequencing, do not differentiate between actively growing, dormant, or dead microbes [7]. Moreover, observed associations between the presence of certain microbial communities and CRS may not be causative [1,7,8].

Indeed, prolonged inflammation may also alter a local microenvironment, secondarily impacting the microbiome composition [8]. Therefore, although understanding the function of microbial communities and their interactions appears promising in developing new therapeutic strategies, the potential of direct manipulation of the microbiome as an effective treatment modality remains unclear [1,7].

Appropriate medical therapy for CRS includes nasal saline rinses and corticosteroids, while the use of antibiotics is controversial [1,2]. Antibiotics may reduce microbial diversity, increase the abundance of pathogenic species, and induce antibiotic resistance [4,16,17,18,19]. Thus, there is growing interest in alternative treatments that can affect the sinonasal microbiome [4]. Among them, probiotics, which contain live strains of bacteria with potentially beneficial effects on the composition of microbial communities, are gaining increasing attention, as they show potential for both passive and active inhibition of pathogens and for modulating host immune response [8,20]. However, to date, the evidence regarding the efficacy of probiotics in CRS is very limited [1,8]. For this reason, the EPOS2020 steering group advised against the use of probiotics in the treatment of patients with CRS. Therefore, further research on probiotics, evaluating their impact on microbial communities, is particularly important.

In this study, we aimed to assess the influence of probiotic rinses in patients suffering from primary chronic rhinosinusitis (CRS), granulomatosis with polyangiitis (GPA), and nasal septal perforation (NSP) on the composition of nasal microbiota using 16S rRNA sequencing.

## 2. Materials and Methods

This prospective, randomized, controlled study was conducted on patients treated in the Otolaryngology Department in Warsaw, Poland, from 2017 to 2021. The inclusion criteria comprised adult patients diagnosed with nasal mucosal diseases, categorized into three groups: chronic rhinosinusitis (CRS), granulomatosis with polyangiitis (GPA), and nasal septal perforation (NSP). Exclusion criteria were cystic fibrosis, primary ciliary dyskinesia, pregnancy, severe lung, heart, or kidney disease, use of oral probiotics, use of intranasal probiotics in the last 6 months, sinus surgery in the last 6 months, antibiotic therapy in the last 2 months, and lack of consent to participate in the study. Patients who did not attend the follow-up evaluation were also excluded.

The study was approved by the Bioethics Committee of the Medical University of Warsaw (decision number KB/198/2015). The study was performed in accordance with the ethical standards of the Declaration of Helsinki and its later amendments.

Within each diagnostic category, patients were randomly assigned to either a study group, which underwent nasal rinsing with a probiotic solution, or a control group, which rinsed the nose with a saline solution. We acknowledge that our study did not employ formal randomization. Instead, participants were assigned to either the study or control group in an alternating sequence (e.g., the first participant was assigned to the study group, the second to the control group, and so on). Although this method does not constitute true randomization, we took care to ensure that there were no significant differences between the groups in key baseline characteristics, including age, SNOT-22 score, and Lund–Kennedy score, as reported in the manuscript. Based on these considerations, we believe the risk of allocation bias was minimized. The group assignments were carried out by the principal investigator.

The study employed a single-blind design: participants were unaware of their group assignments, whereas the researchers were informed of the intervention allocations. This approach was intended to reduce performance bias among participants. To further ensure the reliability of our findings, we used standardized assessment protocols and objective measurement criteria throughout data collection.

Patients in the study group were given capsules containing the following bacteria per 1 g of product:

- *Lactobacillus plantarum* AMT4 1 × 10^10^ cfu/g;
- *Lactobacillus plantarum* AMT14 1 × 10^10^ cfu/g;
- *Bifidobacterium animalis* AMT30 1 × 10^10^ cfu/g.

The capsules also contained sodium chloride and small amounts of xylitol.

In contrast, patients in the control group received capsules containing sodium chloride.

All patients were provided with a nasal rinsing bottle with a marked capacity of 100 mL and instructed to dissolve two capsules in 100 mL of lukewarm, boiled water to prepare the solution. Ultimately, the rinsing solution in the study group contained:

- *Lactobacillus plantarum* AMT4 2 × 10^8^ cfu/mL;
- *Lactobacillus plantarum* AMT14 2 × 10^8^ cfu/mL;
- *Bifidobacterium animalis* AMT30 2 × 10^8^ cfu/mL;
- A 0.9% sodium chloride solution;
- A 0.2% xylitol solution.

The rinsing solution in the control group contained a 0.9% sodium chloride solution. All patients were instructed to perform nasal rinsing twice daily for one month.

All patients filled out the SNOT-22 questionnaire and had an endoscopic examination performed to assess the nasal cavity with a Lund–Kennedy score.

The nasal swab was collected prior to nasal rinsing and the day after the final nasal rinse. If patients rinsed their noses on the day of the visit, the appointment was rescheduled to the following day.

### 2.1. Metagenomic Analysis

Metagenomic analysis of bacterial and archeal populations was carried out by targeting the hypervariable V3-V4 region of the 16S rRNA gene, with taxonomic classification conducted at the levels of the kingdom, phylum, class, order, family, genus, and species, as previously described [21]. Specific primer sequences (341F and 785R) were used to amplify the target region and prepare the sequencing library. PCR amplification was performed using Q5 Hot Start High-Fidelity 2X Master Mix (New England Biolabs, Ipswich, MA, USA), with reaction conditions as recommended by the manufacturer. Sequencing was carried out on the MiSeq apparatus using the paired-end (PE) technology, 2 × 300nt, and the Illumina v3 kit (Illumina, San Diego, CA, USA). Preliminary data analysis was automatically executed on the MiSeq sequencer using the MiSeq Reporter (MSR) v2.6 software (Illumina). The analysis involved two stages:

1. Automatic sample demultiplexing;
2. Generating FASTQ files containing raw reads.

Subsequent bioinformatic analysis for species-level classification was carried out with the QIIME 2 software package using Silva 138 reference sequences. The DADA2 package was employed to identify biological sequences generated during sequencing and to delineate unique sequences of biological origin, i.e., ASVs (amplicon sequence variants).

The analysis comprised the following stages:

1. Quality assessment of reads:
  - Analysis of the error profile for individual samples and dynamic generation of quality control parameters using the FIGARO tool;
  - Quality control based on the coefficient of the maximum expected errors of the sample.
2. Preprocessing data using the Cutadapt tool:
  - Removal of adapter sequences;
  - Rejection reads shorter than 30 nucleotides.
3. Selection of unique ASV sequences (using the DADA2 package) by the following:
  - Filtering out the sequences containing errors created during the sequencing process (denoising);
  - Merging of paired-end reads to enhance accuracy—the corresponding forward and reverse readings must be combined at later stages of the analysis;
  - Dereplication—merging of identical sequences while preserving the number of springs and quality profiles;
  - Chimera filtering to remove artifacts resulting from incorrect sequence assembly during PCR.
4. Assigning a taxonomy to the ASV sequence based on the Silva reference database using a hybrid approach:
  - Comparison of ASV sequences against the reference database using vsearch for identical reference sequences;
  - Classification of atypical sequences remaining after the previous step using machine learning (*sklearn*).

### 2.2. Principal Coordinates Analysis

Data preprocessing and distance calculation: relative abundance data were loaded from a CSV file (Table S1), with taxa as columns and samples as rows. The relative abundance data of microbial taxa were converted from percentages to proportions. The Bray–Curtis dissimilarity metric was used to calculate pairwise distances between samples. This metric was chosen for its suitability in analyzing compositional microbiome data, as it considers both presence/absence and abundance information while being robust to the “double zero” problem common in sparse microbiome datasets.

Principal coordinates analysis: PCoA was performed on the Bray–Curtis distance matrix using the *scikit-bio* library. This method reduces the high-dimensional microbiome data to principal coordinates, allowing visualization of complex community differences in two-dimensional space. The first two principal coordinates, explaining the highest proportion of variance, were used for visualization.

Visualization and group comparisons: we created three separate PCoA plots to analyze specific comparisons of microbial communities:

- Overall comparison: a comprehensive visualization comparing all four groups (before/after probiotic rinsing in the study group and before/after saline rinsing in the control group).
- Probiotic treatment effect: a focused comparison of microbiome composition before and after rinsing with probiotics in the study group.
- Saline treatment effect: a targeted analysis of microbiome composition before and after rinsing with saline in the control group.

This approach allowed for both a holistic view of all treatment conditions and a more detailed examination of specific treatment effects within each group.

Confidence ellipses: to visualize the spread and overlap of different groups in the PCoA space, we calculated and plotted 95% confidence ellipses (1.96 standard deviations) for each group. These ellipses were filled with a transparent color matching the group’s designated color, allowing for easy visualization of group distributions and potential overlaps.

The principal coordinates analysis (PCoA) code for this study was developed with assistance from GitLab Duo, an AI-powered coding assistant (GitLab Inc., San Francisco, CA, USA, 2025). This includes the implementation of data preprocessing, distance matrix calculation, PCoA computation, and visualization of results. All code was thoroughly reviewed and validated by the authors to ensure accuracy and appropriateness for the specific requirements of this microbiome analysis.

For the analysis of changes in clinical outcomes and microbiome data, we analyzed the relationship between changes in clinical outcomes (SNOT-22 and Lund–Kennedy score) and changes in microbiome composition. Group analysis included calculating changes in clinical scores (SNOT-22 and Lund–Kennedy score) for each patient and calculating changes in microbiome abundance for each bacterial taxon in each patient. Then, correlation analysis was conducted using Spearman correlation between changes in clinical scores and changes in microbiome composition—statistically significant correlations *p* < 0.05.

### 2.3. Statistical Analysis

Statistical analysis was performed using Statistica (version 13.0, StatSoft, Poland). A descriptive analysis was conducted, followed by an assessment of distribution normality using the Shapiro–Wilk test. The Student’s t-test was applied to assess differences for variables following a normal distribution. The Mann–Whitney U test was used to evaluate differences in bacterial occurrence between study groups. A two-sided *p*-value  <  0.05 was considered statistically significant. Spearman rank correlation was used to analyze the relationship between clinical scores (SNOT-22 and Lund–Kennedy) and microbiome abundance data.

## 3. Results

Patients’ characteristics are summarized in Table 1. The study group consisted of 22 patients (15 women and 7 men), whereas the control group included 14 patients (10 women and 4 men). No statistically significant differences were observed between the study and control groups in terms of age, SNOT-22 score, and Lund–Kennedy score. The patients’ characteristics in subgroups are shown in Table 2.

### 3.1. Microbiome Analysis

**Figure 1:**
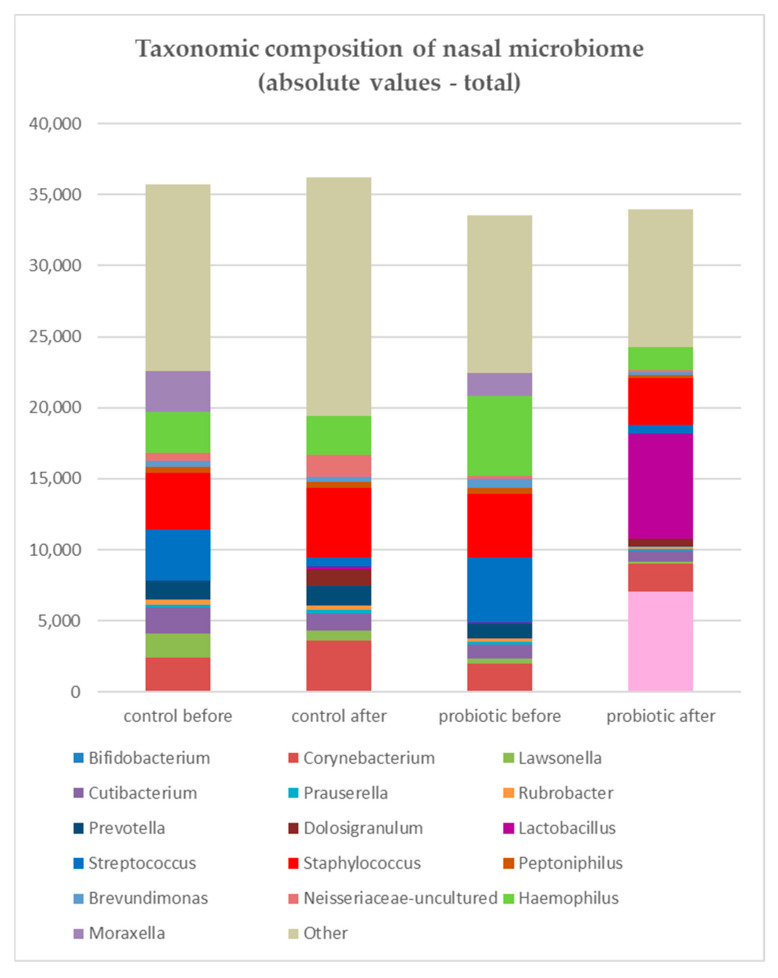
Absolute abundance values of bacterial genera in the nasal microbiome in the study (probiotic) and control (saline) groups. Although *Lactobacillus* and *Bifidobacterium* were visible among other genera after probiotic rinsing, enrichment of the microbiome with probiotic bacteria did not increase the overall bacterial load compared with pre-rinsing levels. In the study group, *Streptococcus* (blue), *Haemophilus* (green), and *Staphylococcus* (red) showed a decrease in total reads, whereas in the control group, only *Streptococcus* decreased while *Staphylococcus* increased.

The results of the microbiome analysis are presented at the genus level. Combined results for both study and control groups are presented in Figure 1, which displays the absolute abundance values and demonstrates that enrichment of the microbiome with probiotic bacteria did not increase the overall bacterial load compared with pre-rinsing levels, while *Lactobacillus* and *Bifidobacterium* were clearly visible among other genera after nasal rinsing with probiotics.

The core microbiome, defined as community composition, included *Staphylococcus*, *Corynebacterium*, *Cutibacterium*, *Prauserella*, *Rubrobacter,* and *Brevundimonas*. *Staphylococcus*, *Corynebacterium,* and *Cutibacterium* were represented at higher abundances, while *Prauserella*, *Rubrobacter*, and *Brevundimonas* were occurring at lower abundances (around 1%). *Streptococcus*, *Haemophilus*, and *Moraxella* were common in the microbiome but varied in prevalence among patients.

**Figure 2:**
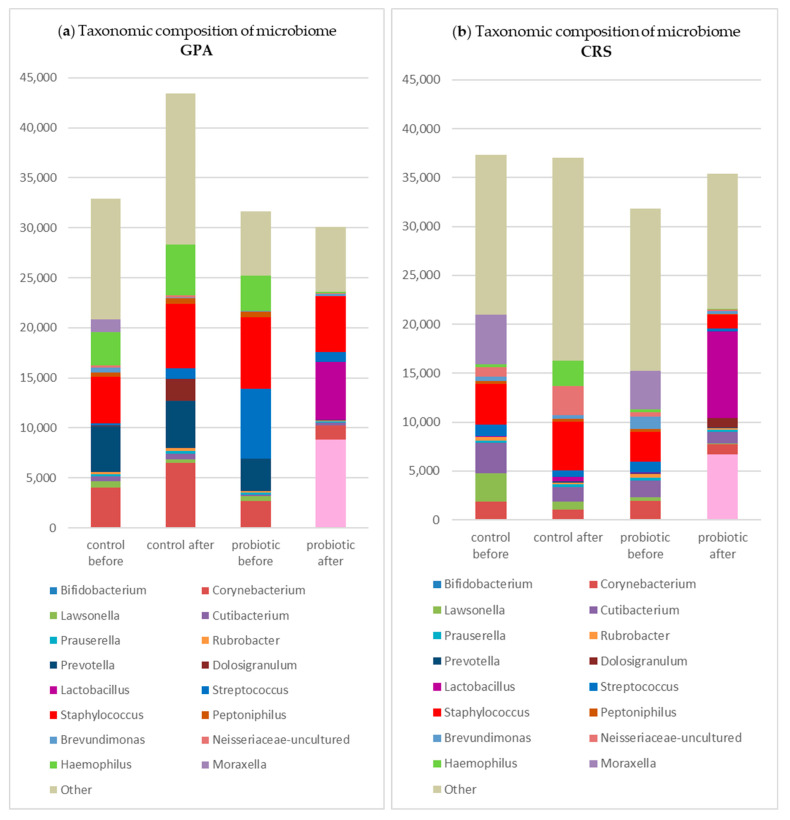

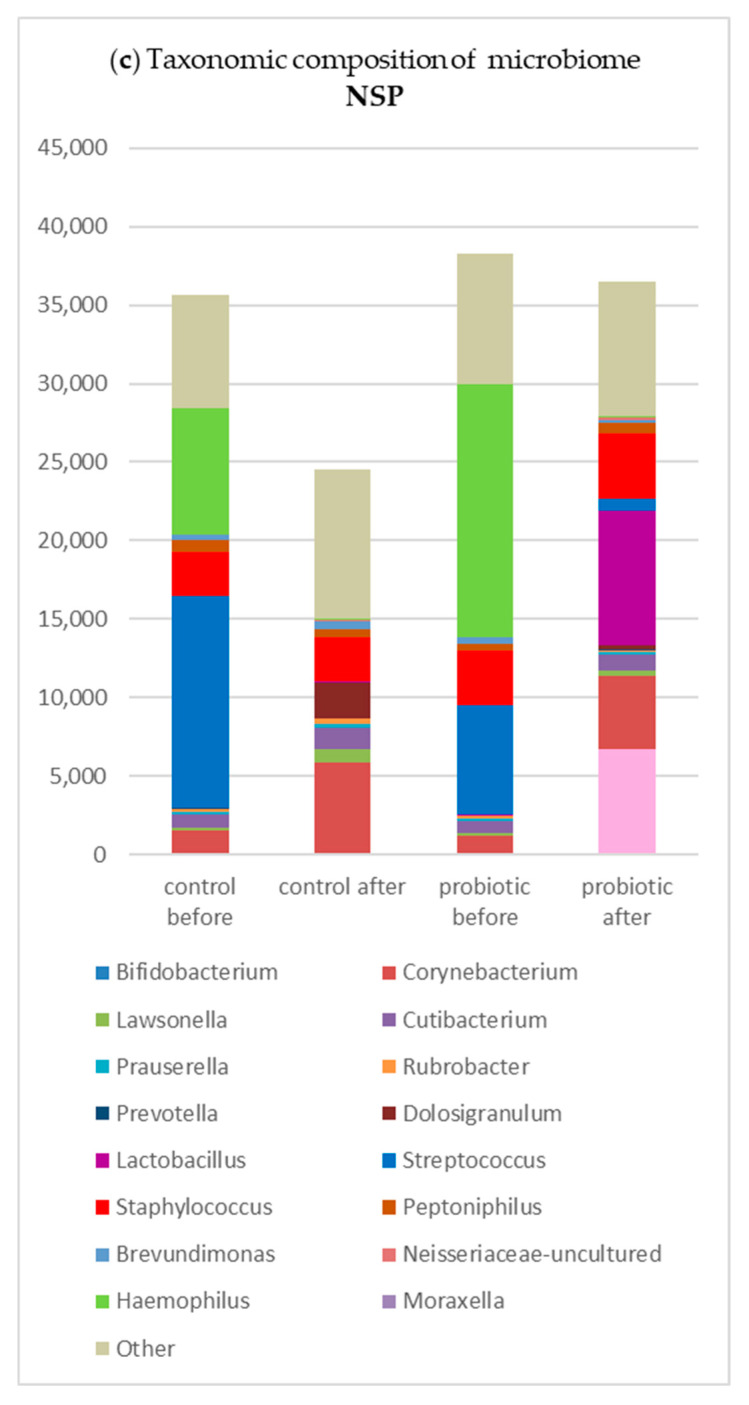
Changes in the taxonomic composition of the nasal microbiome (in absolute values) in subgroups in the study and control groups: (**a**) in GPA patients; (**b**) in CRS patients; (**c**) in NSP patients.

In the study group, after rinsing, *Streptococcus*, *Hemophilus*, and *Staphylococcus* showed a decrease in total reads, whereas in the control group, only *Streptococcus* decreased while *Staphylococcus* increased in terms of mean abundance (Figure 2).

The results for subgroups in the study group are detailed in Table 3 and Table 4.

**Figure 3:**
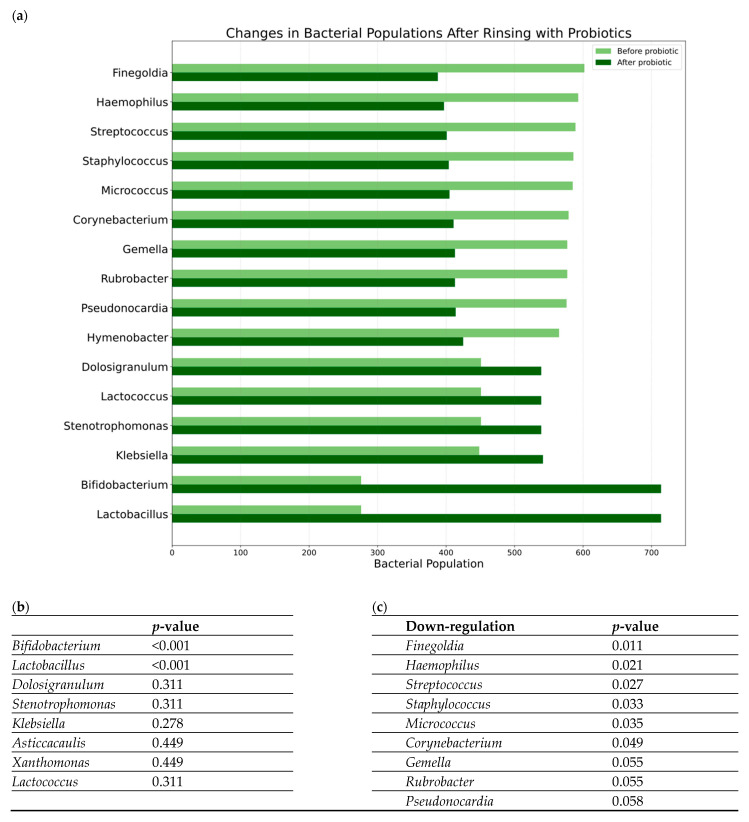
Changes in bacterial populations before and after rinsing with probiotics in the study group: (**a**) a bar chart summarizing the observed changes in the genera abundances; (**b**,**c**) results of the statistical analysis of the changes before and after rinsing with probiotics (*p*-values in Mann–Whitney U test) for genera that decreased their total reads (**b**) and genera that increased their total reads (**c**).

Overall, 435 genera were downregulated, and 108 were upregulated. Notably, among the most abundant co-colonizers were *Staphylococcus*, *Streptococcus*, and *Haemophilus*. After one month of nasal rinsing with probiotics, a significant decreases in the abundance of *Finegoldia* (*p* = 0.010), *Haemophilus* (*p* = 0.020), *Streptococcus* (*p* = 0.027), *Staphylococcus* (*p* = 0.033), *Micrococcus* (*p* = 0.035), *Corynebacterium* (*p* = 0.049), *Gemella* (*p* = 0.055), *Rubrobacter* (*p* = 0.055), and *Pseudonocardia* (*p* = 0.058) genera were observed (*p*-values > 0.05 are not statistically significant). Conversely, there was an obvious increase in the abundance of probiotic species *Lactobacillus plantarum* and *Bifidobacterium animalis*. Moreover, an increase in the genera *Dolosigranulum* and *Stenotrophomonas* was observed, although these observations did not reach statistical significance. Changes in bacterial populations before and after rinsing with probiotics in the study group are presented in Figure 3.

Comparisons between the probiotic and saline groups revealed no statistically significant differences before rinsing, whereas post-rinsing differences are detailed in Table 5.

**Figure 4:**
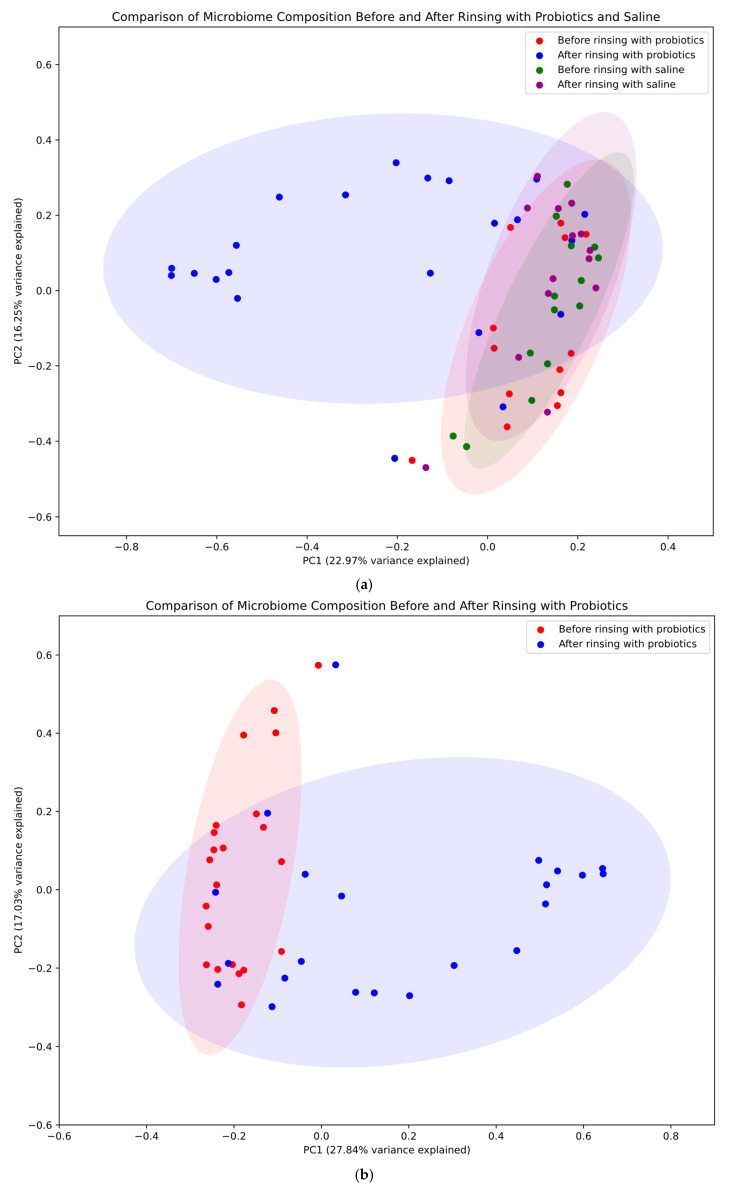

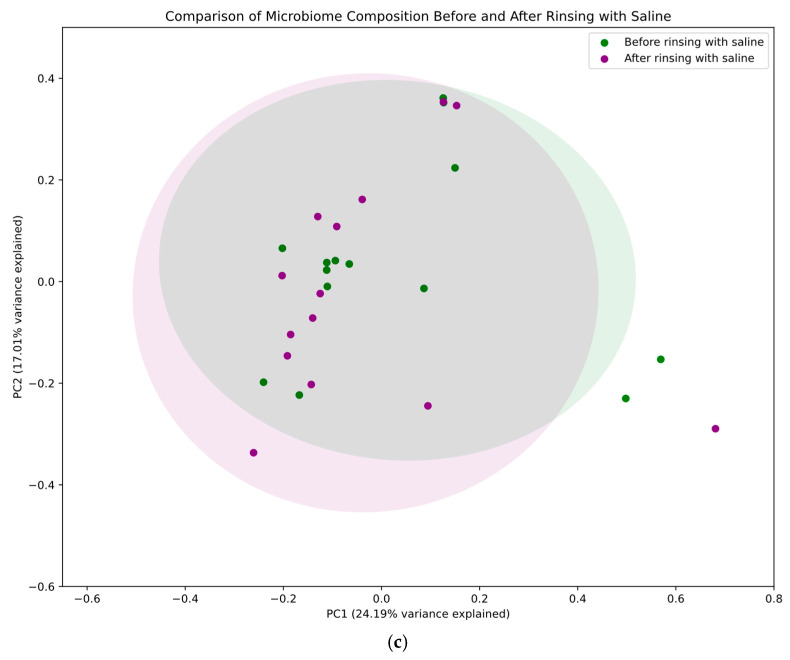
Principal coordinates analysis (PCoA). Four conditions were compared: before and after rinsing with probiotics (study group—red and blue, respectively) and before and after rinsing with saline (control group—green and violet, respectively). PC1 and PC2 denote the first and second principal coordinates, respectively. PC1 (x-axis) represents the axis of greatest variation, while PC2 (y-axis) captures the next highest variance not explained by PC1. These coordinates, derived from the Bray–Curtis dissimilarity matrix of all study samples, are annotated with the percentage of total variation explained (in brackets). This information is critical for interpreting the extent to which the two-dimensional plot represents the overall high-dimensional variation, with higher percentages indicating that observed patterns likely reflect genuine biological differences. (**a**) Microbial community across different treatment groups and time points—the figure for all groups shows the biggest changes to the community when rinsing with probiotics. (**b**) Changes in bacterial populations before and after rinsing with probiotics. (**c**) Changes in bacterial populations before and after rinsing with saline—only minor shifts in the bacterial community were observed in the control group.

A principal coordinates analysis (PCoA) was performed to visualize and compare microbial community structures across treatment groups and time points. The results are presented in Figure 4, which consists of three panels showing different aspects of the microbiome analysis. Panel A provides a comprehensive comparison of all four conditions: before and after rinsing with probiotics in the study group (red and blue, respectively) and before and after rinsing with saline in the control group (green and purple, respectively). In this overall analysis, the first principal coordinate (PC1) accounted for 22.97% of the total variance, while the second principal coordinate (PC2) explained 16.25%. Panel B focuses specifically on changes in bacterial populations before and after rinsing with probiotics, highlighting the substantial shifts in microbial community structure in the study group. When analyzing only the probiotic treatment group, PC1 and PC2 explained 27.84% and 17.03% of the variance, respectively. Panel C illustrates changes in bacterial populations before and after rinsing with saline, demonstrating only minor shifts in the bacterial community in the control group. In this control group analysis, PC1 and PC2 captured 24.19% and 17.01% of the variance, respectively. These percentages indicate that the two-dimensional representations capture a substantial portion of the data’s variation across all analyses. The 95% confidence ellipses (1.96 standard deviations) were used to visualize the distribution and potential overlap between the different groups.

### 3.2. Clinical Outcome and Microbiome Results

The correlations between the clinical outcome and microbiome results were analyzed for all genera and a part of the results—the statistically significant correlations are presented in Table 6.

## 4. Discussion

There is considerable variation in the results of studies investigating the sinonasal microbiome. The systematic review of studies using the 16S rRNA sequencing revealed a high average number of 1587 taxa in healthy controls, with a range of 911–2330 across studies [22]. Notably, the bacterial and fungal microbiome of the respiratory tract is likely dynamic and experiences natural shifts in diversity over time due to changing environmental influences and host factors. Despite this variability, a small core microbial community that persisted throughout the two-year sampling period has been identified, comprising the bacterial genera *Corynebacterium*, *Propionibacterium,* and *Staphylococcus*, as well as the fungal genus *Malassezia restricta* [1,23]. In a large study by Paramasivan et al., the core sinus microbiome in healthy individuals was dominated by five genera: *Corynebacterium*, *Staphylococcus*, *Streptococcus*, *Moraxella*, and *Haemophilus* [13]. The authors divided this core microbiome into two tiers based on the observed significant disparity between the organisms. The first tier, composed of *Corynebacterium* and *Staphylococcus*, was nearly ubiquitous among healthy controls, leading the authors to the conclusion that these genera are likely key commensals in maintaining sinus physiology [11,13]. Similarly, Hoggard et al. found bacterial communities from non-CRS subjects to be variable but consistently dominated by the genera *Corynebacterium* and *Staphylococcus* [24]. The second tier in the study by Paramasivan et al. was formed by *Streptococcus*, *Haemophilus*, and *Moraxella*, representing the most common abundant co-colonizers. These organisms may, therefore, be commensals with relatively low bacterial burden within the sinonasal mucosa, while a dysbiosis state with their over-representation may lead to acute infectious or inflammatory processes [13]. Indeed, next-generation sequencing (NGS) studies have conclusively demonstrated the presence of previously considered “pathogenic” bacteria in healthy sinuses [8]. Interestingly, in our study group with three different rhinologic entities, the core microbiome was dominated by *Staphylococcus* and *Corynebacterium* but also included *Cutibacterium*, *Prauserella*, *Rubrobacter*, and *Brevundimonas*. Bacteria considered “pathogenic” were the unstable part of the microbiome.

A meta-analysis by Wagner Mackenzie et al. exploring differences in sinonasal bacterial community composition between healthy and CRS-affected subjects identified the most abundant bacteria across all subjects as *Staphylococcus*, *Propionibacterium*, *Corynebacterium*, *Streptococcus*, and an unclassified lineage of *Actinobacteria* [23]. Interestingly, the authors found significantly lower bacterial diversity and increased abundance of the genus *Corynebacterium* to be associated with CRS. Moreover, increased relative abundance and diversity of other members belonging to the phylum *Actinobacteria* and members from the genus *Propionibacterium* differentiated healthy and CRS patients. In addition, the study highlighted the potential importance of the genera *Burkholderia* and *Propionibacterium* as “gatekeepers”, suggesting that their presence may be important in maintaining a stable sinonasal microbiome [23]. In our previous study, we observed that *Propionibacterium* (*Cutibacterium*) was even lower in patients with systemic disease (GPA) than in patients with chronic rhinosinusitis [21]. In a systematic review by Gomez-Garcia et al., out of 24 studies that compared the results from CRSwNP patients with those from non-CRS subjects, six studies reported no significant differences in bacterial species, number of different isolates obtained, total bacterial load, or measures of both alpha diversity (intra-community diversity) and beta diversity (intersample variability) [4].

In contrast, the most common differences reported in the remaining studies included a decrease in alpha diversity; an underabundance of the bacterial genera *Corynebacterium* and *Dolosigranulum*; an overabundance of different bacteria of the phylum *Proteobacteria*, such as *Hemophilus*, *Moraxella*, or *Pseudomonas*; and differences in the abundance of specific *Firmicutes* species [4]. Similarly, in our study, we observed a predominance of *Staphylococcus*, *Streptococcus*, *Hemophilus*, and *Corynebacterium* in individuals with chronic disease without acute exacerbation. Notably, *Haemophilus* and *Moraxella* may induce epithelial damage and promote host inflammatory pathways, whereas lipopolysaccharides present in cell walls of *Pseudomonas* and other Gram-negative bacteria may induce persistent mucosal inflammation and subsequent tissue remodeling.

Although Gomez-Garcia et al. found the results obtained for bacteria from the phylum *Firmicutes* to be controversial, they noted a potential role of *Staphylococcus aureus* colony formation in the sustained inflammatory response associated with CRS [4]. Nevertheless, the presence of *S. aureus* in the sinuses of both healthy and CRS individuals indicates that it may exhibit either commensal or pathogenic behavior depending on its overall abundance and interaction with other microbiota, with its “unchecked” expansion contributing to inflammation [8]. This hypothesis is supported by an in vitro study demonstrating a biphasic response in an anti-inflammatory cytokine IL-10 and pro-inflammatory cytokine TNF-alpha based on *S. aureus* bacterial load: a relatively low bacterial load induced an IL-10 peak with minimal TNF-alpha, while at a higher bacterial load, IL-10 decreased and TNF-alpha increased [25]. In the present study, we observed a decrease in the abundance of *Staphylococcus* (also at the species level: *Staphylococcus aureus*) after probiotic rinses, but the result did not reach statistical significance.

The decrease in *Corynebacterium* and *Dolosigranulum* abundance observed in CRS by Gomez-Garcia et al. was attributed to their role as commensals in the airway mucosa, potentially exerting a protective function by modulating the innate immune response [4]. On the other hand, as previously mentioned, a meta-analysis by Wagner Mackenzie et al. [23] identified the *Corynebacterium* phylotype as a potential biomarker of CRS-associated sinonasal microbiota. Of note, while *Corynebacteria* are typically regarded as nasal commensals, certain species, such as *C. tuberculostearicum,* have been implicated in CRS pathogenesis [13,26].

Interestingly, Yan et al. [27] reported that in healthy nasal mucosa, *Staphylococcus aureus* and *Corynebacterium accolens* co-occur, whereas *Corynebacterium pseudodiphtheriticum* is more prevalent in non-carriers of *S. aureus*, suggesting a potential cooperative interaction between *S. aureus* and *C. accolens* and a competitive interaction between *S. aureus* and *C. pseudodiphtheriticum*. These findings are consistent with the results of their in vitro studies, revealing that S. aureus growth was enhanced in proximity to *C. accolens* while inhibited in proximity to *C. pseudodiphtheriticum*. In contrast, Menberu et al. [28] found that *Corynebacterium accolens* exhibited antimicrobial activity against both planktonic and biofilm growth of *S. aureus*. In our study, we observed a significant difference in *Corynebacterium* abundance between the study and control groups after one month of nasal rinsing, but the analysis at the level of species was inconclusive due to poor discrimination at this level.

In an NGS study of CRSwNP patients and healthy individuals, Uzunoğlu et al. [29] observed that the predominant bacterial phyla in all groups were *Firmicutes*, *Proteobacteria*, and *Actinobacteria*, while the predominant genera were *Staphylococcus*, *Corynebacterium*, and *Sphingomonas*. *Corynebacterium* was identified as the differentiating taxon for the control group, whereas *Streptococcus*, *Moraxella*, *Rothia*, *Micrococcus*, *Gemella*, and *Prevotella* were characteristic of CRSwNP patients [29]. *Malassezia* was the predominant fungal genus across all groups. Furthermore, *Staphylococcus* demonstrated a statistically significant negative correlation with *Dolosigranulum*, while *Corynebacterium* showed a positive correlation with *Anaerococcus* and negative correlations with *Neisseria*, *Prevotella*, *Fusobacterium*, and *Peptostreptococcus* [29]. Koeller et al. found significantly enriched *Flavobacterium, Pseudomonas, Pedobacter, Porphyromonas, Stenotrophomonas*, and *Brevundimonas* in the CRSsNP samples compared to the healthy controls [30]. In a study involving patients undergoing endoscopic sinus surgery (ESS), Jain et al. found that the minority taxon *Finegoldia* decreased in abundance following ESS and in patients with reduced total bacterial burden but increased in patients with higher symptom scores [31]. Notably, in our study, *Finegoldia* was the taxon that exhibited a significant decrease in abundance after probiotic rinses (*p* = 0.011).

Importantly, the CRS microbiome is typically characterized by decreased diversity [23,26,32]. This finding, in the presence of a similar overall bacterial burden, indicates the role of dysbiosis in CRS, defined by shifts in the relative abundance of taxa, with a potential expansion of pathogenic bacteria and a reduction in the commensal populations [8]. Commensal bacteria play an essential role in maintaining the balance of a healthy microbiome by competing with pathogens for mucosal surface, cell surface receptors, and nutrients. Moreover, they secrete antimicrobial compounds, such as hydrogen peroxide, lactic acid, and bacteriocins, and modulate the host immune response [8,14].

Several studies have investigated the effects of various treatment modalities on the sinonasal microbiome [2,4,8,17,31]. The first-line treatment in CRS is medical therapy, with surgery reserved for refractory cases [1,8]. Appropriate medical therapy includes topical and oral corticosteroids and saline rinses, while the benefit of concurrent antibiotics use remains ambiguous [1,2]. Nevertheless, several studies found no significant alteration in the sinonasal microbiome of CRSwNP patients after the use of topical steroids and nasal saline irrigation [2,4,17,31]. Therefore, it is hypothesized that different mechanisms, such as the reduction in inflammatory mediators and improving mucociliary clearance, contribute to their efficacy in improving sinonasal symptoms [4]. In contrast, antibiotic therapy has been associated with reduced microbial diversity [4,16,17] and increased *S. aureus* abundance [16], and their excessive use may promote antibiotic resistance within the microbiome [4,18]. Thus, there is growing interest in alternative treatments capable of modulating the sinonasal microbiome.

One such modality could be the use of probiotics [8]. Their proposed mechanisms of action include both passive and active inhibition of pathogens, augmentation of the epithelial barrier, secretion of antimicrobial compounds, and modulation of the host immune response [8]. Probiotic bacteria, particularly members of the genus *Lactobacillus*, enhance or protect epithelial barrier function in vitro and in animal models via modulation of tight junctions. Changes in tight junction proteins mediated by TLR2 signaling have been observed in an in vitro model of human epithelium following the administration of *L. plantarum* [33]. Similarly, *Bifidobacterium animalis* has demonstrated potential as a probiotic to help maintain epithelial integrity. In vitro, it displayed a positive tolerance toward simulated digestive tract conditions and adhered well to the intestinal epithelium. In vivo, it restored tight junctions, enhanced systemic and intestinal IgA secretion, and improved the intestinal microbiota balance [34].

Although much attention has been given to probiotics in research on the gut microbiome, evidence for their use in CRS is scarce [1,8]. Two murine studies have shown a protective effect of locally administered probiotics against pathogen-mediated sinonasal inflammation [8,26,35], and some studies suggest that probiotics are able to improve mucosal barrier function [36]. Abreu et al., having identified *Lactobacillus sakei* as a potentially protective species in comparative microbiome analyses of CRS patients and healthy controls, found reduced *C. tuberculostearicum* abundance and a protective effect on sinus epithelium in the animals co-instilled with *L. sakei* when compared to animals infected with *C. tuberculostearicum* alone [26]. Similarly, Cleland et al. observed that animals receiving nasal inoculants containing a combination of *Staphylococcus aureus* and *Staphylococcus epidermidis* had significantly lower goblet cell counts compared to those receiving *S. aureus* alone, thereby mitigating the typical pro-inflammatory effects of the pathogenic bacteria [35]. However, the translation of in vitro and animal study outcomes to human populations is challenging, and human studies to date fail to provide clear evidence of the efficacy of probiotics in improving the microbiome characteristics in CRS patients [1,8,37].

In a randomized controlled trial by Mukerji et al. involving 77 CRS patients, oral administration of *Lactobacillus rhamnosus* caused significant improvement in SNOT-22 scores at 4 weeks but not at 8 weeks after treatment, and no significant changes in symptom frequency were observed [37]. In contrast, two studies assessing the effect of oral *Enterococcus faecalis* administration reported a decrease in the frequency of acute exacerbations of CRS [38] and a reduction in the frequency and duration of episodes of recurrent acute sinusitis [39], although its impact on microbiota was not assessed. Mårtensson et al. found no significant changes in SNOT-22 scores, microbiome, or levels of inflammatory markers (IL-6, IL-8, TNF-, IL-8, and MPO) in nasal lavage of CRSsNP patients following the administration of a nasal spray containing 13 lactic acid bacteria (*Lactobacillus* and *Bifidobacterium*) [40]. Endam et al. reported improvements in sinus symptoms, SNOT-22 scores, and mucosal POSE (peri-operative sinus endoscopy) scores after irrigation with *Lactococcus lactis* W136, while no difference in olfactory function assessed by UPSIT-40 was observed [41]. In our previous study, we also observed a reduction in symptoms based on the SNOT-22 questionnaire (*p* = 0.002 in GPA, not significant—in rhinitis/rhinosinusitis) after one month of nasal rinsing with *Lactobacillus plantarum* and *Bifidobacterium animalis*. In nasal endoscopy, the reduction in the intensity of changes in GPA and CRS was statistically significant. In addition, patients with primary rhinitis/rhinosinusitis also experienced a reduction in nasal mucosa irritation and crusting in the nose (*p* < 0.05) [42]. Regarding microbiota composition, only an increased abundance of *Dolosigranulum pigrum*, a bacteria potentially beneficial in the upper airways [43], was observed, with no other significant microbiome changes, including no significant changes in alpha and beta diversities.

In this study, we found that the probiotic rinsing resulted in significant changes in the bacterial community, particularly among the genera *Hemophilus*, *Streptococcus*, *Staphylococcus*, and *Corynebacterium*. An interesting observation was the increase in the abundance of *Dolosigranulum*, a bacterial genus potentially beneficial in the upper airways, following nasal rinsing with probiotics, although this change was not statistically significant. These findings, in conjunction with the existing literature, suggest potential benefits of probiotic rinsing.

Our study investigated bacterial community changes in three different rhinologic entities (GPA, CRS, NSP). We decided to analyze these three subgroups both separately and collectively for several reasons. Initially, we analyzed all the data in subgroups, which significantly reduced the statistical power. We decided to analyze the material collectively because all patients had previously undergone treatment with antibiotics—both systemic and topical—due to exacerbations of primary disease, which we consider a common factor for the three groups, significantly influencing the microbiome composition. Moreover, we found no statistically significant differences between the study group and the control group in this study. Finally, we assumed that the selected probiotics would exert a similar effect on the bacterial population across all groups. Patients included in our study had already undergone treatment for their primary disorder and were in the stable phase of the disease without exacerbation. Interestingly, enriching the nasal microbiome with probiotics altered the relative abundance of certain genera while maintaining the total bacterial load. Principal coordinates analysis (PCoA) graphically depicted these changes, capturing a higher proportion of the total variation in the dataset compared with previous studies [44] and delineating the differences between saline and probiotic rinsing groups.

Our initial 1-month timeframe was selected to assess the immediate effects of probiotic nasal rinses on the nasal microbiota in patients with primary chronic rhinosinusitis (CRS), granulomatosis with polyangiitis (GPA), and nasal septal perforation (NSP). To date, only a limited number of studies have investigated the role of topical probiotics in the treatment of CRS, and none have evaluated their use in patients with GPA. Existing studies in this area have also utilized relatively short observation periods. For instance, in the study conducted by Endam et al., 16S rRNA microbiome analysis was performed after just 14 days, with additional microbial assessment using bacterial culture after 28 days [41]. Mårtensson et al. evaluated microbial changes after 2 weeks, using only bacterial cultures [40]. In contrast, Passali et al. investigated a 3-month regimen of probiotic nasal sprays and extended the observation period to 12 months to monitor infection episodes; however, microbiome analyses were not performed [45]. We intend to design future studies with extended follow-up periods to comprehensively assess both short- and long-term effects of probiotic nasal therapy.

Genera like *Finegoldia* and *Peptoniphilus* showed a significant positive correlation with LK score changes (worse outcome). Notably, *Finegoldia* abundance significantly decreased after probiotic rinsing (Figure 3, *p* = 0.011). This reinforces the potential pathogenic role of *Finegoldia*, as suggested by its decrease following the potentially beneficial probiotic intervention and its association here with poorer endoscopic outcomes. The decrease observed post-probiotics might contribute to the clinical improvements seen in some patients. The correlation of *Peptoniphilus* (another anaerobe) with worse LK scores further supports the idea that shifts in anaerobic bacterial populations might be relevant to endoscopic findings.

The association of *Moraxella* with worse symptoms in the saline group aligns with its known role as a potential respiratory pathogen. The negative correlation of *Rubrobacter* and *Alteribacillus* with symptoms, or *Micrococcus* with LK score, in the saline group, is intriguing and might point towards potentially protective roles for these bacteria that become apparent during the non-specific washout effect of saline, although their overall abundance might be low. It is interesting to contrast *Micrococcus*—its increase correlated with better endoscopy in the saline group, but its overall abundance significantly decreased after probiotic rinsing (Figure 3, *p* = 0.035). These findings, while correlative, highlight the complex interplay between specific microbial taxa and distinct aspects of clinical outcomes in inflammatory nasal diseases and underscore that different therapeutic approaches may engage different microbial pathways.

Considering the choice of bacterial strains such as *Lactobacillus plantarum* AMT4/AMT14 in our study, we need to underline that the probiotic properties of bacteria are mostly strain-specific and not species-specific. Strains can exhibit differences in various traits; they may differ in observable characteristics like size, shape, growth patterns, or the presence of specific proteins or antigens; and in behavior, strains within the same species can behave differently in certain environments or respond differently to treatments [46]. Understanding strain-level diversity is crucial in various fields, including microbiome research, where strain-specific effects on host health are becoming increasingly recognized; in probiotic research, the efficacy of probiotic strains can be highly specific, meaning that not all strains of a species are equally beneficial [47]. Our selection of probiotic strains was based on a literature analysis of potential pathogens causing upper respiratory tract infections and on the results of proprietary studies on the antagonistic properties of probiotic bacteria from our collection against selected pathogens. In order to confirm the antagonistic properties of probiotic strains against pathogens, a series of in vitro tests of probiotic bacteria were conducted against selected pathogens, *including Clostridium difficile* ATTC BAA 1870, *Clostridium perfringens* NCTC 8678, *Klebsiella pnemoniae* ATCC BAA 1706, *Pseudomonas aeruginosa* NCIMB 8626, *Staphylococcus aureus* NCIMB 12702, *Streptococcus anginosus* ATCC 33397, *Streptococcus pyogenes* ATCC 12344, and *Candida tropicalis* ATCC 1369. Based on the results, strains that presented the strongest antagonistic properties against selected pathogens were selected, and they did not show antagonistic properties against other probiotic strains.

Our study has several limitations. The heterogeneous study population—including patients with CRS, GPA, and NSP—may complicate interpretation, although it also suggests the potential applicability of probiotics across various sinonasal inflammatory conditions. The small sample size, particularly within subgroups, may have limited the statistical power to detect significant microbiome changes or correlations with clinical outcomes such as SNOT-22 and Lund–Kennedy scores. Additionally, natural temporal microbiome fluctuations, the short duration of probiotic treatment, and the lack of long-term follow-up preclude assessment of sustained clinical effects. Larger, long-term studies are warranted.

## 5. Conclusions

Although the mechanism of probiotic action theoretically makes them a promising treatment method, the lack of convincing evidence for their efficacy in the treatment of sinonasal mucosa inflammation has led the EPOS2020 steering group to advise against the use of probiotics in the treatment of CRS [1]. Nonetheless, a key aspect of this study was the assessment of probiotic rinsing on microbial community composition, which demonstrated its capacity to alter the microbiota when compared with saline rinsing.

## Figures and Tables

**Table 1:** Comparison of patient characteristics between the study and control groups *.

	Study Group *n* = 22 Mean (SD)	Control Group *n* = 14 Mean (SD)	*p*-Value
**Age**	44.0 (12.1)	40.4 (12.6)	0.393
**Symptoms and endoscopic scores before treatment**			
**SNOT-22 total score**	34.3 (20.3)	33.9 (20.4)	0.955
**Lund–Kennedy score**	5.4(2.5)	5.2 (2.5)	0.800
**Symptoms and endoscopic scores after treatment**			
**SNOT-22 total score**	27.2 (18.3)	29.9 (21)	0.681
**Lund–Kennedy score**	3.7 (1.8)	5 (2.2)	0.056

* All data are presented as mean (SD) and were compared with Student’s *t*-tests.

**Table 2:** Characteristics of patients in subgroups *.

	GPA		NSP		CRS	
	Study Group	Control Group	Study Group	Control Group	Study Group	Control Group
	*n* = 7	*n* = 4	*n* = 6	*n* = 3	*n* = 9	*n* = 7
Age	42.6 ± 11.7	40.5 ± 15.7	43.3 ± 13.5	50.3 ± 14.0	45.6 ± 12.9	35.4 ± 8.7
SNOT-22 before treatment	36.1 ± 22.0	31.5 ± 25.3	46.6 ± 21.3	53.0 ± 19.8	26.1 ± 16.3	25.7 ± 14.5
SNOT-22 after treatment	25.3 ± 20.1	23.0 ± 26.1	32.3 ± 19.5	44.7 ± 13.0	25.2 ± 17.7	27.6 ± 20.2
Lund–Kennedy score before treatment	7.1 ± 2.7	5.9 ± 4.2	4.8 ± 1.7	4.7 ± 2.1	4.5 ± 2.3	5.2 ± 1.7
Lund–Kennedy score after treatment	6.9 ± 5.4	5.1 ± 3.5	2.9 ± 1.6	5.0 ± 0.0	3.5 ± 1.1	5.0 ± 2.1

* All data are presented as mean (SD).

**Table 3:** Mean abundance of genera in subgroups before probiotic rinsing: (**a**) in granulomatosis with polyangiitis (GPA); (**b**) in chronic rhinosinusitis (CRS); (**c**) in nasal septal perforation (NSP).

(a)										
GPA	1	2	3	4	5		6	7		Mean
**Staphylococcus**	65.0%	9.4%	7.4%	58.6%	12.1%		26.5%	8.9%		26.8%
**Haemophilus**	0.0%	24.3%	2.9%	0.5%	0.0%		54.7%	0.0%		11.8%
**Streptococcus**	0.2%	0.7%	0.2%	3.9%	74.9%		2.1%	0.0%		11.7%
**Corynebacterium**	16.4%	1.0%	37.4%	4.6%	0.3%		0.4%	1.6%		8.8%
**Unrecognised bacteria**	8.6%	1.8%	3.6%	12.6%	9.1%		13.7%	0.1%		7.1%
**Prevotella**	0.0%	32.7%	11.6%	3.1%	0.0%		0.0%	0.0%		6.8%
**Alteribacillus**	0.3%	0.0%	0.0%	0.3%	0.2%		0.0%	17.4%		2.6%
**Prauserella**	1.4%	0.1%	0.1%	0.4%	0.4%		0.3%	10.2%		1.8%
**Rubrobacter**	0.3%	0.2%	0.0%	0.5%	0.8%		0.5%	9.3%		1.7%
**Peptostreptococcus**	0.0%	5.6%	5.3%	0.1%	0.0%		0.0%	0.0%		1.6%
**Peptoniphilus**	0.8%	2.7%	7.0%	0.0%	0.0%		0.0%	0.0%		1.5%
**Cutibacterium**	0.6%	0.2%	0.3%	1.6%	0.2%		0.1%	7.2%		1.5%
**Campylobacter**	0.0%	0.4%	1.8%	0.3%	0.0%		0.0%	6.5%		1.3%
**Proteus**	0.0%	2.1%	6.4%	0.0%	0.0%		0.0%	0.0%		1.2%
**Micrococcus**	0.1%	0.0%	0.0%	0.3%	0.0%		0.1%	7.0%		1.1%
**Lawsonella**	1.5%	4.7%	0.7%	0.0%	0.0%		0.0%	0.0%		1.0%
**Fusobacterium**	0.0%	6.8%	0.0%	0.0%	0.0%		0.0%	0.0%		1.0%
**(b)**										
**CRS**	**8**	**9**	**10**	**11**	**12**	**13**	**14**	**15**	**16**	**Mean**
**Unrecognized bacteria**	48.58%	31.03%	0.05%	40.82%	9.77%	34.32%	27.74%	22.23%	26.57%	26.79%
**Staphylococcus**	2.24%	7.17%	16.21%	12.69%	26.54%	1.79%	5.62%	1.76%	41.70%	12.86%
**Corynebacterium**	1.08%	18.94%	7.03%	15.90%	9.02%	4.18%	3.17%	0.30%	2.63%	6.92%
**Brevundimonas**	1.20%	1.68%	3.78%	3.22%	0.74%	0.41%	1.14%	46.27%	0.34%	6.53%
**Cutibacterium**	7.09%	7.15%	9.53%	2.35%	20.21%	1.77%	1.56%	0.50%	0.41%	5.62%
**Moraxella**	0.00%	0.00%	0.00%	0.00%	0.00%	42.28%	0.00%	0.00%	0.00%	4.70%
**Streptococcus**	1.80%	0.66%	7.70%	0.39%	6.11%	4.38%	0.88%	0.00%	5.43%	3.04%
**Rubrobacter**	4.63%	1.58%	0.37%	0.44%	0.34%	0.45%	1.36%	4.65%	1.08%	1.65%
**Lawsonella**	0.00%	5.24%	0.00%	4.41%	4.51%	0.00%	0.00%	0.00%	0.00%	1.57%
**Neisseriaceae;** **Genus uncultured**	0.00%	0.00%	0.48%	1.06%	9.95%	0.00%	0.96%	0.00%	0.00%	1.38%
**Pseudomonas**	0.00%	0.00%	0.15%	0.00%	0.00%	0.00%	1.59%	10.46%	0.00%	1.36%
**Prauserella**	0.58%	1.44%	0.58%	0.46%	0.11%	0.30%	0.64%	6.43%	1.23%	1.31%
**Peptoniphilus**	0.00%	5.13%	0.45%	0.50%	0.25%	0.75%	0.68%	0.00%	2.27%	1.11%
**Anaerococcus**	1.57%	2.97%	1.89%	0.16%	0.56%	0.55%	1.01%	0.00%	0.00%	0.97%
**Enhydrobacter**	0.37%	0.18%	0.11%	0.00%	2.05%	0.07%	5.67%	0.00%	0.23%	0.96%
**Chryseobacterium**	6.33%	0.00%	0.00%	0.00%	0.69%	0.00%	1.32%	0.00%	0.00%	0.93%
**Rothia**	0.30%	0.34%	3.68%	0.17%	0.41%	2.68%	0.37%	0.00%	0.10%	0.89%
**Paracoccus**	3.15%	0.00%	1.44%	0.00%	0.18%	0.00%	2.47%	0.00%	0.00%	0.80%
**Haemophilus**	0.00%	0.29%	2.78%	0.03%	1.72%	0.88%	0.05%	0.00%	1.43%	0.80%
**Phenylobacterium**	2.35%	0.43%	0.45%	0.50%	0.15%	0.08%	0.00%	2.78%	0.00%	0.75%
**Brochothrix**	0.00%	0.00%	0.00%	4.20%	0.00%	0.00%	2.03%	0.00%	0.42%	0.74%
**Alteribacillus**	1.14%	0.38%	0.52%	0.76%	0.12%	0.23%	0.31%	2.34%	0.60%	0.71%
**Micrococcus**	1.44%	0.00%	0.00%	0.13%	0.42%	0.00%	2.90%	0.72%	0.00%	0.62%
**Sphingomonas**	0.33%	0.30%	2.34%	0.00%	0.24%	0.12%	1.66%	0.00%	0.00%	0.56%
**Finegoldia**	0.00%	2.24%	0.59%	0.99%	0.24%	0.16%	0.45%	0.00%	0.31%	0.55%
**(c)**										
**NSP**	**17**	**18**	**19**	**20**	**21**	**22**				**Mean**
**Haemophilus**	0.3%	42.8%	94.9%	0.0%	0.1%	0.2%				23.1%
**Staphylococcus**	32.5%	1.6%	0.3%	59.2%	11.0%	6.6%				18.5%
**Streptococcus**	11.2%	41.9%	1.2%	0.5%	38.1%	0.9%				15.7%
**Unrecognised bacteria**	27.8%	3.6%	1.7%	0.0%	16.9%	25.1%				12.5%
**Corynebacterium**	1.2%	2.5%	0.1%	9.1%	7.0%	3.2%				3.8%
**Cutibacterium**	2.0%	1.2%	0.0%	4.0%	3.8%	5.9%				2.8%
**Brevundimonas**	0.4%	0.2%	0.0%	5.4%	1.8%	1.6%				1.6%
**Peptoniphilus**	0.6%	0.4%	0.0%	0.6%	4.7%	0.7%				1.2%
**Rubrobacter**	3.8%	0.1%	0.1%	0.3%	0.3%	0.5%				0.8%
**Sphingomonas**	0.4%	0.0%	0.0%	0.8%	0.4%	3.1%				0.8%
**Anaerococcus**	0.0%	0.4%	0.0%	0.2%	3.5%	0.5%				0.8%
**Prauserella**	2.2%	0.3%	0.1%	0.7%	0.4%	0.8%				0.7%
**Micrococcus**	0.8%	0.1%	0.0%	1.1%	0.5%	1.6%				0.7%
**Kocuria**	1.1%	0.0%	0.0%	1.5%	0.0%	0.8%				0.6%
**Finegoldia**	0.0%	0.5%	0.0%	0.0%	1.6%	1.0%				0.5%
**Rothia**	1.5%	0.0%	0.6%	0.2%	0.0%	0.8%				0.5%

**Table 4:** Mean abundance of genera in subgroups after probiotic rinse: (**a**)—in granulomatosis with polyangiitis (GPA); (**b**)—in chronic rhinosinusitis (CRS); (**c**)—in nasal septal perforation (NSP).

(a)										
GPA	a	b	c	d	e	f		g		Mean
**Bifidobacterium**	42.6%	31.3%	4.8%	24.0%	27.2%	11.5%		51.5%		27.5%
**Lactobacillus**	14.0%	49.1%	2.1%	47.8%	10.5%	9.4%		32.0%		23.6%
**Staphylococcus**	6.7%	15.2%	68.4%	1.5%	1.1%	10.9%		3.9%		15.4%
**Unrecognized** **bacteria**	24.0%	1.4%	3.9%	20.5%	43.6%	16.3%		0.0%		15.7%
**Streptococcus**	0.4%	0.0%	0.1%	0.0%	0.1%	42.4%		4.5%		6.8%
**Corynebacterium**	0.8%	0.7%	15.8%	0.4%	3.9%	1.6%		0.8%		3.4%
**Escherichia-Shigella**	6.3%	0.0%	0.0%	0.0%	0.0%	0.0%		0.0%		0.9%
**Cutibacterium**	0.8%	0.9%	0.0%	0.8%	0.5%	0.3%		1.5%		0.7%
**Brevundimonas**	0.1%	0.0%	0.1%	0.2%	1.4%	2.2%		0.1%		0.6%
**Neisseriaceae;** **Genus_uncultured**	0.0%	0.0%	0.0%	3.8%	0.2%	0.0%		0.0%		0.6%
**Prauserella**	0.7%	0.0%	0.1%	0.2%	0.3%	1.5%		0.9%		0.5%
**Lactococcus**	0.0%	0.0%	0.0%	0.0%	3.4%	0.0%		0.0%		0.5%
**Rubrobacter**	0.4%	0.0%	0.0%	0.3%	0.2%	1.2%		0.9%		0.4%
**Haemophilus**	0.0%	0.0%	0.0%	0.0%	2.3%	0.0%		0.0%		0.3%
**Klebsiella**	0.0%	0.0%	2.0%	0.0%	0.0%	0.0%		0.0%		0.3%
**(b)**										
**CRS**	**h**	**i**	**j**	**k**	**l**	**m**	**n**	**o**	**p**	**mean**
**Unrecognized bacteria;**	42.36%	1.74%	41.98%	1.54%	48.66%	0.68%	30.82%	29.33%	25.10%	24.69%
**Lactobacillus**	0.00%	33.83%	15.10%	53.29%	1.84%	50.03%	0.18%	7.99%	6.33%	18.73%
** Bifidobacterium**	0.00%	21.18%	11.39%	42.07%	4.43%	42.50%	0.41%	5.47%	4.29%	14.64%
** Staphylococcus**	6.13%	1.47%	8.21%	0.02%	2.04%	4.88%	3.64%	7.73%	9.68%	4.87%
** Cutibacterium**	11.90%	0.24%	0.63%	0.02%	16.00%	0.67%	2.89%	7.28%	0.53%	4.46%
**Corynebacterium**	4.91%	0.14%	1.10%	0.00%	1.48%	0.22%	14.24%	14.69%	0.85%	4.18%
**Dolosigranulum**	0.00%	0.00%	0.00%	0.00%	4.15%	0.00%	28.93%	0.00%	0.00%	3.68%
**Pseudomonas**	0.00%	31.41%	0.33%	0.02%	0.00%	0.00%	0.00%	0.00%	0.77%	3.61%
** Marinomonas**	0.00%	0.00%	0.00%	0.00%	0.00%	0.00%	0.00%	0.11%	20.01%	2.24%
**Brochothrix**	0.00%	0.00%	0.00%	0.00%	0.00%	0.00%	0.05%	9.66%	7.09%	1.87%
**Anaerococcus**	1.00%	0.02%	0.19%	0.00%	9.83%	0.00%	0.38%	0.00%	0.50%	1.32%
**Brevundimonas**	2.96%	0.16%	0.84%	0.18%	1.50%	0.04%	1.90%	1.15%	0.58%	1.03%
**Streptococcus**	2.32%	0.00%	3.50%	0.20%	0.00%	0.00%	1.09%	0.47%	1.28%	0.99%
**Klebsiella**	0.00%	8.40%	0.00%	0.00%	0.09%	0.00%	0.00%	0.00%	0.00%	0.94%
**Campylobacter**	8.31%	0.00%	0.00%	0.00%	0.00%	0.00%	0.00%	0.00%	0.00%	0.92%
**Leuconostoc**	0.00%	0.00%	0.00%	0.00%	0.12%	0.00%	0.00%	0.71%	6.65%	0.83%
**Prauserella**	1.69%	0.16%	0.56%	0.00%	1.92%	0.00%	0.60%	0.55%	1.30%	0.75%
**Rubrobacter**	0.86%	0.05%	0.58%	0.03%	1.44%	0.08%	0.55%	0.26%	1.09%	0.55%
**(c)**										
**NSP**	**q**	**r**	**s**	**t**	**u**	**w**				**mean**
**Lactobacillus**	2.62%	4.88%	1.36%	46.59%	61.34%	0.50%				19.55%
**Bifidobacterium**	25.37%	10.72%	0.77%	35.88%	30.86%	0.00%				17.27%
**Haemophilus**	0.00%	0.04%	95.93%	0.00%	0.00%	1.15%				16.19%
** Unrecognized bacteria**	29.94%	8.68%	1.24%	8.65%	0.00%	14.85%				10.56%
**Staphylococcus**	3.88%	22.71%	0.18%	3.55%	0.48%	13.04%				7.31%
**Corynebacterium**	0.00%	35.72%	0.12%	0.21%	0.05%	4.69%				6.80%
**Cutibacterium**	2.49%	1.68%	0.16%	0.63%	0.19%	9.09%				2.37%
**Streptococcus**	3.61%	0.65%	0.02%	0.14%	0.00%	7.91%				2.05%
**Anaerococcus**	0.00%	1.01%	0.00%	0.07%	0.01%	8.28%				1.56%
**Peptoniphilus**	0.00%	0.61%	0.00%	0.03%	0.00%	8.44%				1.51%
**Finegoldia**	0.00%	0.08%	0.00%	0.00%	0.00%	6.66%				1.12%
**Neisseria**	0.00%	0.00%	0.00%	0.00%	5.34%	0.89%				1.04%
**Prauserella**	4.68%	0.18%	0.07%	0.12%	0.08%	0.41%				0.92%
**Family Bacillaceae**	4.37%	0.13%	0.00%	0.00%	0.00%	0.04%				0.76%
**Rubrobacter**	3.55%	0.31%	0.05%	0.03%	0.05%	0.25%				0.71%
**Rothia**	0.77%	0.34%	0.00%	0.32%	0.00%	2.73%				0.69%
**Lawsonella**	0.00%	0.00%	0.03%	0.07%	0.00%	3.52%				0.60%
**Phenylobacterium**	2.81%	0.00%	0.00%	0.18%	0.00%	0.26%				0.54%

**Table 5:** Genera showing significant differences after treatment (study group—probiotic rinsing vs. control group—saline rinsing). No statistically significant differences were observed between groups before treatment *.

Genus	*p*-Value *
*Bifidobacterium*	<0.001
*Lactobacillus*	<0.001
*Gemella*	0.003
*Nocardioides*	0.027
*Finegoldia*	0.027
*Tepidimonas*	0.027
*Methylobacterium-Methylorubrum*	0.035
*Haemophilus*	0.045
*Deinococcus*	0.053
*Cutibacterium*	0.057
*Neisseria*	0.057

* All data were compared using the Mann–Whitney U test.

**Table 6:** SNOT-22 and Lund–Kennedy score changes and their correlations with microbiome (**a**) in the probiotic rinsing group and (**b**) in the saline rinsing group (Spearman’s rank correlation).

(a)						
Genus	SNOT22 Change Correlation	SNOT22 Change *p* Value	LK Change Correlation	LK Change *p* Value	SNOT22 Significant	LK Significant
Coprococcus	0.584	0.005	0.145	0.519	True	False
Ruminococcus	0.496	0.022	0.367	0.093	True	False
Agathobacter	0.461	0.035	0.072	0.751	True	False
Roseburia	0.435	0.049	0.072	0.751	True	False
Dietzia	−0.435	0.049	−0.304	0.169	True	False
Rathayibacter	−0.435	0.049	−0.191	0.394	True	False
Haliangium	−0.435	0.049	−0.191	0.394	True	False
Prosthecobacter	−0.435	0.049	−0.191	0.394	True	False
Janibacter	−0.506	0.019	−0.109	0.629	True	False
Cloacibacterium	−0.512	0.018	0.275	0.216	True	False
Faecalibacterium	0.366	0.103	0.424	0.049	False	True
Finegoldia	0.206	0.371	0.489	0.021	False	True
Peptoniphilus	0.110	0.635	0.513	0.015	False	True
Xanthomonas	0.033	0.886	0.436	0.042	False	True
Lawsonella	−0.025	0.914	0.466	0.029	False	True
Luteimonas	−0.203	0.378	−0.433	0.044	False	True
Terrimonas	−0.343	0.128	−0.479	0.024	False	True
**(b)**						
**Genus**	**SNOT22** **Change** **Correlation**	**SNOT22** **Change** ***p* Value**	**LK** **Change** **Correlation**	**LK** **Change** ***p* Value**	**SNOT22** **Significant**	**LK** **Significant**
Pseudoxanthomonas	0.662	0.014	0.397	0.160	True	False
Veillonella	0.610	0.027	−0.265	0.360	True	False
Moraxella	0.566	0.044	0.425	0.130	True	False
Pseudopropio-nibacterium	0.561	0.047	0.194	0.506	True	False
Tannerella	−0.568	0.043	−0.372	0.191	True	False
Rubrobacter	−0.576	0.040	−0.474	0.087	True	False
Alteribacillus	−0.781	0.002	−0.394	0.163	True	False
Negativicoccus	0.372	0.211	0.543	0.045	False	True
Exiguobacterium	0.345	0.248	0.543	0.045	False	True
Altererythrobacter	0.345	0.248	0.543	0.045	False	True
Vibrionimonas	0.133	0.666	0.543	0.045	False	True
Abiotrophia	0.018	0.953	0.662	0.010	False	True
Phenylobacterium	−0.139	0.650	−0.571	0.033	False	True
Lautropia	−0.163	0.594	−0.574	0.032	False	True
Micrococcus	−0.363	0.223	−0.632	0.015	False	True
Pontibacter	−0.372	0.211	−0.614	0.019	False	True

## Data Availability

Data are contained within the article or Supplementary Materials.

## Supplementary Materials

The following supporting information can be downloaded at: https://www.mdpi.com/article/10.3390/jcm14103341/s1, Table S1: Microbiome: Genus—all samples.

## Abbreviations

The following abbreviations are used in this manuscript:

16S rRNA	16S ribosomal RNA
ASV	Amplicon sequence variants
CRS	Chronic rhinosinusitis
CRSwNP	Chronic rhinosinusitis with nasal polyps
CRSsNP	Chronic rhinosinusitis without nasal polyps
EPOS2020	European Position Paper on Rhinosinusitis and Nasal Polyps 2020
ESS	Endoscopic sinus surgery
GPA	Granulomatosis with polyangiitis
NGS	Next-generation sequencing
NSP	Nasal septum perforation
PE	Paired-end
PC	Principal coordinate
PCoA	Principal coordinates analysis
PCR	Polymerase chain reaction
